# Prevalence of Inflammatory Pathways Over Immuno-Tolerance in Peripheral Blood Mononuclear Cells of Recent-Onset Type 1 Diabetes

**DOI:** 10.3389/fimmu.2021.765264

**Published:** 2022-01-04

**Authors:** Aritania Sousa Santos, Edécio Cunha-Neto, Nelson Vinicius Gonfinetti, Fernanda Bernardi Bertonha, Pauline Brochet, Aurelie Bergon, Carlos Alberto Moreira-Filho, Christophe Chevillard, Maria Elizabeth Rossi da Silva

**Affiliations:** ^1^ Laboratorio de Carboidratos e Radioimunoensaios LIM 18, Faculdade de Medicina, University of Sao Paulo Hospital of Clinics, São Paulo, Brazil; ^2^ Laboratory of Immunology, Heart Institute, School of Medicine, University of São Paulo, São Paulo, Brazil; ^3^ Instituto Castro de Medicina, São Paulo, Brazil; ^4^ Department of Pediatrics, School of Medicine, University of Sao Paulo, Sao Paulo, Brazil; ^5^ Aix Marseille Université, Inserm, TAGC Theories and Approaches of Genomic Complexity, INSERM, UMR_1090, Marseille, France

**Keywords:** type 1 diabetes (T1D), peripheral blood mononucleated cells (PBMC), gene expression, immune activation, immune regulation, LTF, DEFA, AREG

## Abstract

**Background:**

Changes in innate and adaptive immunity occurring in/around pancreatic islets had been observed in peripheral blood mononuclear cells (PBMC) of Caucasian T1D patients by some, but not all researchers. The aim of our study was to investigate whether gene expression patterns of PBMC of the highly admixed Brazilian population could add knowledge about T1D pathogenic mechanisms.

**Methods:**

We assessed global gene expression in PBMC from two groups matched for age, sex and BMI: 20 patients with recent-onset T1D (≤ 6 months from diagnosis, in a time when the autoimmune process is still highly active), testing positive for one or more islet autoantibodies and 20 islet autoantibody-negative healthy controls.

**Results:**

We identified 474 differentially expressed genes between groups. The most expressed genes in T1D group favored host defense, inflammatory and anti-bacterial/antiviral effects (*LFT, DEFA4, DEFA1, CTSG, KCNMA1*) and cell cycle progression. Several of the downregulated genes in T1D target cellular repair, control of inflammation and immune tolerance. They were related to T helper 2 pathway, induction of FOXP3 expression (*AREG*) and immune tolerance (*SMAD6*)*. SMAD6* expression correlated negatively with islet ZnT8 antibody. The expression of *PDE12*, that offers resistance to viral pathogens was decreased and negatively related to ZnT8A and GADA levels. The increased expression of long non coding RNAs MALAT1 and NEAT1, related to inflammatory mediators, autoimmune diseases and innate immune response against viral infections reinforced these data

**Conclusions:**

Our analysis suggested the activation of cell development, anti-infectious and inflammatory pathways, indicating immune activation, whereas immune-regulatory pathways were downregulated in PBMC from recent-onset T1D patients with a differential genetic profile.

## Introduction

Type 1 diabetes (T1D) is a heterogeneous autoimmune disease caused by the pancreatic islet infiltration of auto-reactive T and B cells, macrophages, natural killer and dendritic cells (insulitis), associated with several inflammatory and immunologic mechanisms, cytokine and chemokine pathways that increase and perpetuate the damage (1).

Islet autoantibodies are the gold standard humoral markers of active beta cell autoimmunity, usually present in the preclinical phase of the disease, being important prognostic factors to the development of T1D. However, they do not inform accurately when and which individuals will progress to overt diabetes (1).

The immunological alterations in human islets and the mechanisms underlying progressive beta cell destruction are still not fully known (1). This requires knowledge on gene expression alterations in both pancreatic and in immune effector cells, justifying the search for new mechanisms and additional biomarkers.

Peripheral blood samples are readily accessible and can bring insights about dysregulated pathways related to the process. Even though they may not exactly mirror the aggression-taking place in the pancreas, they provide valuable information about the immunological status of the patients with T1D (2, 3). Interactions between these tissues were supported by expanded clones of islet-antigen-reactive CD4+T cells in peripheral blood of subjects with T1D (3) and by the presence of PBMC receptors of several cytokines and chemokines expressed in islet of T1D patients exposed to cytokines (4). Activation of the immune system, mainly mediated by Interferon (IFN) gamma, has important prognostic and follow-up implications for identifying individuals at risk for T1D (4–8). Analysis of peripheral blood mononuclear cells (PBMCs) have also defined transcriptional alterations associated to several autoimmune diseases like systemic lupus erythematosus (SLE) (9), multiple sclerosis (MS) (9), rheumatoid arthritis (RA) (10), Crohn’s disease (CD) (11) and ulcerative colitis (UC) (11). They contributed to the knowledge of specific inflammatory gene expression patterns associated with ERK, MAPK, IFN, NFkB pathways, providing insights into the pathogenic mechanisms of autoimmune processes (12).

However, transcriptomic studies of PBMCs of recent Caucasian T1D patients yielded conflicting information related to activation of innate and adaptive immune genes, present in some (3, 5, 13) but not all evaluations (4, 14), perhaps due to the different patient populations, gene sets and analysis techniques. Environmental and genetic predisposition, age of patients and diabetes duration, as long-term diabetes exhibited chronic inflammation, possibly contributed to divergences among studies.

The large gene expression variations among individuals justify more research to define their immunological functions and potential involvement in T1D development. Complementary contributions can come from populations with different genetic component like the highly admixed Brazilian population with T1D patients, presenting major contributions coming from European ancestry (0.77), followed by African (0.15) and Amerindian ancestries (0.073). Different frequency of risk polymorphisms for T1D in our population (15) in relation to those referred as Caucasians, could also elicit a distinct gene expression in T1D.

Therefore, we investigated if the gene expression profiling of PBMCs from recent-onset T1D patients, a condition in which the autoimmune process is still highly active could add evidence to new protective and pathogenic pathways. A microarray covering 60,901 probe sets and their associations with clinical and laboratorial data were provided.

Our results indicated the involvement of pathways favoring cell proliferation, inflammation and defense against bacterial and virus infection over tolerance. Different environmental and genetic factors could have contributed to downregulation of molecules related to immunomodulation like AREG and SMAD6, not previously reported in T1D.

## Material and Methods

The study was approved by the Research Ethical Committee of Hospital das Clinicas, Faculdade de Medicina, Universidade de Sao Paulo (Cappesq 11601), following guidelines in the Declaration of Helsinki. Written informed consents were obtained from every subject or an authorized representative.

### Study Design and Patients

The study enrolled two clinical groups, matched for age, sex and body mass index: T1D group (20 patients with recent-onset T1D (≤ 6 months from diagnosis) according to American Diabetes Association criteria (16), expressing at least one islet autoantibody; and Control group (20 islet autoantibody-negative healthy controls without family history of inflammatory or autoimmune diseases or concomitant medications). Exclusion criteria included other types of diabetes, use of medications except insulin or an active or presumed infection in the previous ten days before blood collection

Demographic data and clinical characteristics of the selected individuals are described in **Table 1**. As expected, in comparison with Controls, T1D patients had higher HbA1c and glucose levels, higher titers of autoantibodies against glutamic acid decarboxylase (GAD65A), tyrosine phosphatase (IA-2A) and zinc transporter 8 (ZnT8A) and higher frequency of HLA-DR3 or –DR4 alleles. Considering self-reported skin color, there was a higher frequency of non-white in the T1D group.

**Table 1:** Clinical characteristics of patients with type 1 diabetes (T1D) and healthy controls.

	Healthy Controls	T1D	*P*
Number	20	20	
Age (years)	13.2 (10.3-17.6)	13.2 (9.1-23.5)	0.9100
Female/Male (n)	13F/7M	12F/8M	1.0000
Glycemia (mg/dL)	78.0 (75.2-86.0)	105.5 (82.5-202.5)	0.0053
HbA1c (%)	5.2 (5.1-5.4)	9.2 (6.6-11.0)	< 0.0001
HbA1c (mmol/mol)	33.3 (32.2-35.5)	77.0 (48.6-96.7)	
GAD65A (IU/mL)	0.1 (0.0-0.6)	213.0 (44.4-250.0)	< 0.0001
Znt8A (IU/mL)	0.0 (0.0-2.2)	882.7 (91.5-1.000)	< 0.0001
IA-2A (IU/mL)	2.3 (1.9-3.1)	274.4 (33.7-400.0)	< 0.0050
Diabetes duration (years)	——	0.22 (0.14-0.48)	
HLA-DR3 or –DR4	6/14	13/5	0.0217
White/Non-white	18/2	10/10	0.0058
Body Mass Index (kg/m2)	20,50	21,66	0.7985

HLA aleles, HLA-DR*0301, HLA-DR*0401, HLA-DR*0402, HLA-DR*0404.

HLA-DR*0405; GAD65A - glutamic acid decarboxylase antibody.

ZnT8A- Zinc transporter 8 antibody; IA-2A- tyrosine phosphatase antibody.

### Peripheral Blood Mononuclear Cells separation

Peripheral blood samples were collected in BD Vacutainer^®^ CPT™ BD tubes containing sodium citrate, separator gel and Ficoll ™ Hypaque ™ solution. The samples were centrifuged for 30min at 2.300 rpm at room temperature. The pellet was washed with Hanks Buffer (HBSS- Gibco™ -Thermofisher), centrifugated at 1.800 rpm for 10min, lysed with ACK lysing buffer –Gibco™, washed again with the same buffer and centrifuged. The PBMC pellet was re-suspended in RNAlater reagent (Ambion- Thermofisher, USA and stored at freezer -80°C.

### RNA Extraction, Quality, and Integrity

The RNA extraction used the RNeasy Mini Kit (Qiagen- Germany), for 1x10^6^ human cells, according to the manufacturer’s protocol. Total RNA purity was certificated in a NanoDrop ND-2100 spectrophotometer (Thermofisher-USA), evidencing values for the ratio optical density OD260/OD280 superior to 1.8. The RNA integrity was obtained using the RNA 6000 Nano kit on BioAnalyzer 2100 (Agilent- Technologies, USA). All samples presented RNA Integrity Number (RIN) ≥ 8.0, exhibiting the presence of distinct ribosomal bands 28SrRNA and 18SrRNA, with 2:1 intensity ratio, demonstrating that the RNA was intact (**Supplementary Table S1**). After analysis, all samples were stored at -80°C, until used in hybridization experiments.

### Microarray Hybridization and Global Gene Expression

Gene expression profile determination used a 60,901 probe sets microarray (SurePrint G3 Human Gene Expression v3 8x60K Microarray, G4858A-072363- Agilent Technologies, Inc., Santa Clara, CA, USA). The procedures for hybridization, using the fluorescent dye Cy3 followed the manufacturer’s protocol (One Color Microarray-Based Gene Expression Analysis-Low Input Quick Amp Labeling Agilent Technologies, USA). The Agilent One-Color Microarray-based Gene Expression Analysis uses cyanine 3-labelled targets to measure gene expression in experimental and control samples and Oligo dT-Promoter Primer and T7 RNA Polymerase to generate cRNA. The total RNA (100ng) containing 2μL of spike-in control was amplified, fluorescently labeled, and hybridized to microarray chips according to the manufacturer’s instruction. The hybridization process was performed for 17 h at 65°C into the hybridization oven rotator rack at a speed of four rounds per minute. After hybridization, appropriate washing steps followed the manufacturer’s protocol.

The microarray images were captured by the reader Agilent Bundle according to parameters recommended for bio-arrays and extracted by Agilent Feature Extraction software version 10.7.3 for gene expression.

Expression data were loaded into an R-environment using the AgiND package (v2.1.4) (DOI: 10.1186/1755-8794-7-28). Quantile normalization using the normalize Quantile function was performed. All microarray raw data have been deposited in Gene Expression Omnibus (GEO) public database (http://www.ncbi.nlm.nih.gov/geo), a MIAME compliant database, under accession number GSE156035.

### Validation of the DEGs Identified in the Transcriptome

The expression of a subset of five genes was validated by qRT-PCR using TaqMan assays – (Applied Biosystems- Thermofisher – Ca-USA): three upregulated genes, namely: LTF Hs00914334_m1, DEFA4 Hs00157252_m1 and CTSG Hs00175195_m1 and two downregulated genes: AREG- Hs006969-m1 SMAD6 -Hs00178579_m1. The endogenous control was Actin Beta (ACTB) Hs99999903_m1.The gene transcription used the High Capacity kit (Part Number 4368814) – Applied Biosystems- Thermofisher Ca-USA).

The expression levels of the genes evaluated were consistent with the results obtained in the transcriptome

### Biochemical Analyses

Serum fasting glucose levels were determined using an enzymatic colorimetric assay (LABTEST GOD-ANA, SP, Brazil) and glycated hemoglobin (HbA1c) levels by high performance liquid chromatography. Serum levels of the autoantibodies against glutamic acid decarboxylase (GAD65A) and tyrosine phosphatase (IA-2A) were determined by radioimmunoassay (RSR limited, UK; CV < 7%). The normal values for 700 healthy controls (considered three standard deviations, SD) were < 25.0 IU/mL and < 125 IU/mL for GAD65A and IA-2A, respectively. Serum levels of the autoantibodies against Zinc transporter 8 (ZnT8A) were measured by ELISA (KR770-96; Kronus, USA; CV <7%). The normal value of ZnT8A in 321 healthy controls was defined as ≤ 16 IU/mL (considered 3 SD).

### Statistical Analysis

The distributions of demographic data were verified by the Shapiro-Wilk normality test. Numerical variables with parametric and non-parametric distribution were analyzed by unpaired Student’s t- test and Mann-Whitney test (with Dunn’s Multiple Comparison post-test), respectively. The correlations were performed with Pearson’s correlation coefficient or Spearman’s rank correlation coefficient. Qualitative variables were compared using the chi-square test or the Fisher’s exact test, with the statistical package GraphPad Prism. P< 0.05 was considered significant.

### Data and Resource Availability

The data sets have been deposited in GEO public database under accession number GSE156035. They are also available in supplementary data or by the corresponding author upon request.

## Results

### Gene Expression Profile

Gene expression was done on 20 patients with recent-onset T1D and 20 Controls. Transcriptional analysis revealed that among the 60901 probe sets tested on the Agilent microarrays, 526 probes related to 478 genes (transcripts) were differentially expressed (DEGs) in PBMCs from new-onset T1D patients in comparison to healthy controls (**Supplementary Table 2S**).

**Figure 1:**
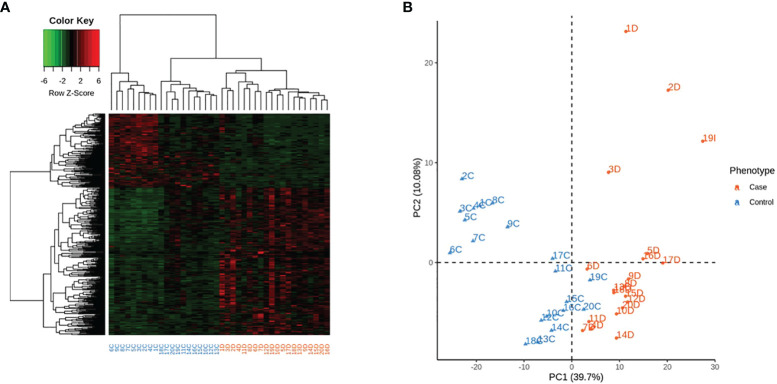
Expression of the 346 differentially expressed genes (DEG) across the 40 samples, according to their phenotype. Control identifiers are written in blue, and case identifiers in orange. **(A)** Complete-linkage hierarchical clustering (HCA) based on spearman correlation. **(B)** First two components of principal component analysis (PCA).

Among the 478 genes, 346 were protein-coding genes recognized by Ingenuity Pathway Analysis (IPA) (**Supplementary Table 3S**) and subjected to further analysis. T1D –RNAs were distinguished by hierarchical clustering and principal component analysis (**Figure 1**).

The T1D group exhibited a higher number of upregulated than downregulated genes (213 vs 133, respectively). The expression of the most up-regulated genes in T1D patients was increased by 2.7- to 12.6 fold change over healthy controls and of the under-expressed genes was decreased by -2.9 to -5.7 fold (**Table 2**). All were included in the analysis of canonical pathways, networks and upstream regulators, providing disease-related patho-biological processes and pathways.

**Table 2:** Expression (#) of top 10 up-regulated and down-regulated genes in patients with T1D group in comparison with health controls.

Expression pattern	Genes	FC(T1D/control)	P value
Up regulated	LFT*	12,559	2,41E-02
	DEFA4	7,786	3,02E-02
	DEFA1	5,704	3,97E-02
	CTSG	4,914	2,87E-02
	ESCO2*	3,595	1,22E-02
	ASAP1-IT1-	2,918	5,15E-03
	MTR*	2,911	1,73E-02
	ZNF66*	2,881	1,03E-02
	KCNMA1*	2,735	4,21E-02
	NEK2*	2,673	2,77E-02
			
Down regulated	AREG*	-5,684	4,92E-02
	KRT86*	-4,647	2,07E-02
	SMAD6	-4,348	3,17E-02
	SLC2A7	-4,029	3,48E-02
	WHRN* (DFNB31)	-3,973	2,98E-02
	CXCL8	-3,89	1,95E-02
	STX19	-3,89	3,15E-02
	NECTIN4	-3,366	4,87E-02
	PDE12	-2,872	2,41E-02
	KIF5A	-2,836	3,78E-02

^#^Expression is shown as fold change (FC) over the control group.

*means more than one probe.

### Molecular and Cellular Functions

The pathways enrichment analysis of the DEGs of PBMCs from patients with recent-onset T1D, revealed as main molecular and cellular functions: cell death and survival, amino acid metabolism, small molecule biochemistry, cell cycle and cellular development.

**Figure 2:**
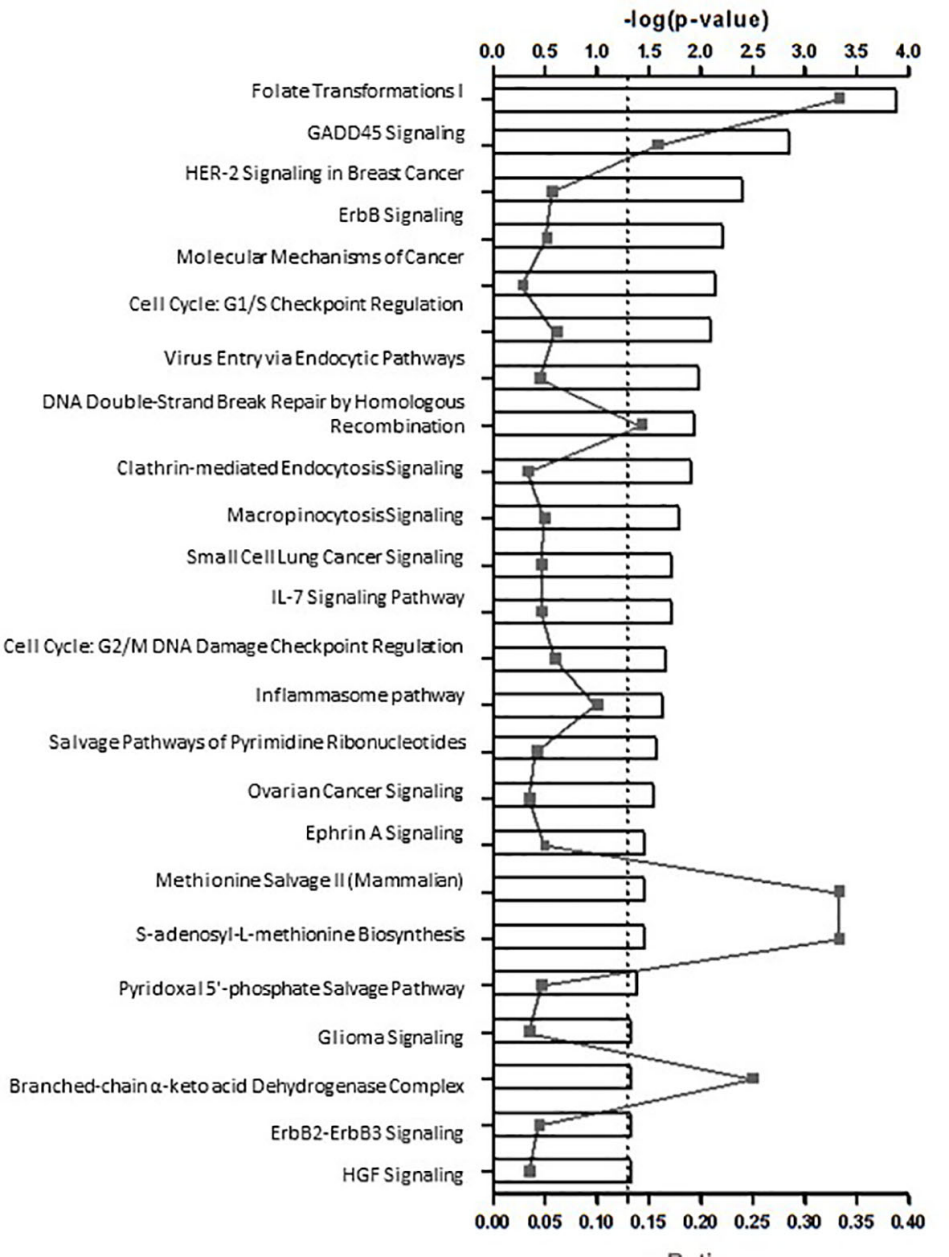
24 potential canonical pathways enriched in patients with autoimmune type 1 diabetes (n=20) in comparison to health controls (n=20).

These functions were represented in the 24 enriched Canonical pathways predicted with a high likelihood (**Figure 2**), comprising cell development, metabolic and inflammatory pathways like: nucleotide synthesis, DNA repair, cell cycle, cancer, metabolism, endocytosis, cytokine signaling and the inflammasome pathway - Innate and adaptive immune responses and defense against bacteria and viruses. The DEGs belonging to these pathways are depicted in **Supplementary Table 4S**.

**Figure 3:**
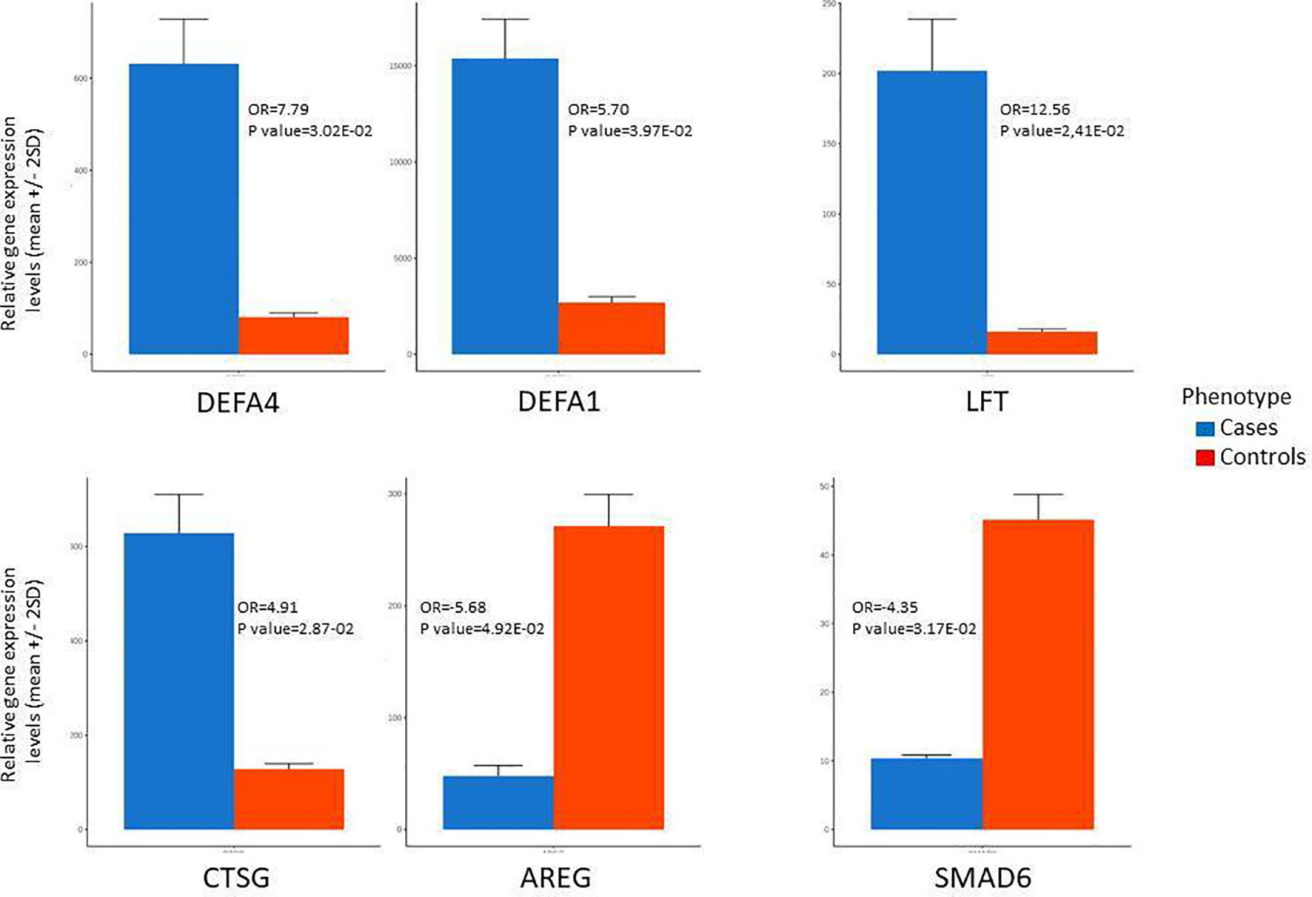
Most differentially expressed genes between patients with type 1 diabetes (cases) and health controls based on microarrays data. AREG, Amphiregulin; CTSG, Cathepsin G; DEFA1, Defensin alpha 1; DEFA4, Defensin alpha 4; KCNMA1, Potassium Large Conductance Calcium-Activated Channel; LTF, Lactoferrin; SMAD6, Mothers Against Decapentaplegic Homolog 6.

The top upregulated and downregulated genes in T1D patients in comparison to controls are in **Table 2**. The most expressed genes in PBMCs from patients with T1D encompassed host defense. Importantly, the four top upregulated genes encoded antimicrobial/pro-inflammatory peptides: *DEFA4, DEFA1, LFT and CTSG* (**Figure 3**)*. KCNMA1*, another upregulated gene, had similar characteristics. Other upregulated genes were directed to the control of genomic stability, cell cycle progression/mitosis, and apoptosis (*ESCO2, ASAP1-IT1, NEK2*) or involved in metabolism (*MTR*) and transcriptional regulation (*ZNF66*).

The most down-modulated genes were related to T helper 2 pathway activation and induction of FOXP3 expression *(AREG*), immune tolerance (SMAD6) (**Figure 3**), response to bacterial and viral infection and inflammation (*CXCL8, PDE12, STX19, DFNB31*), cell transport of glucose and organelles (*KIF5A, SCL2A7*).

**Figure 4:**
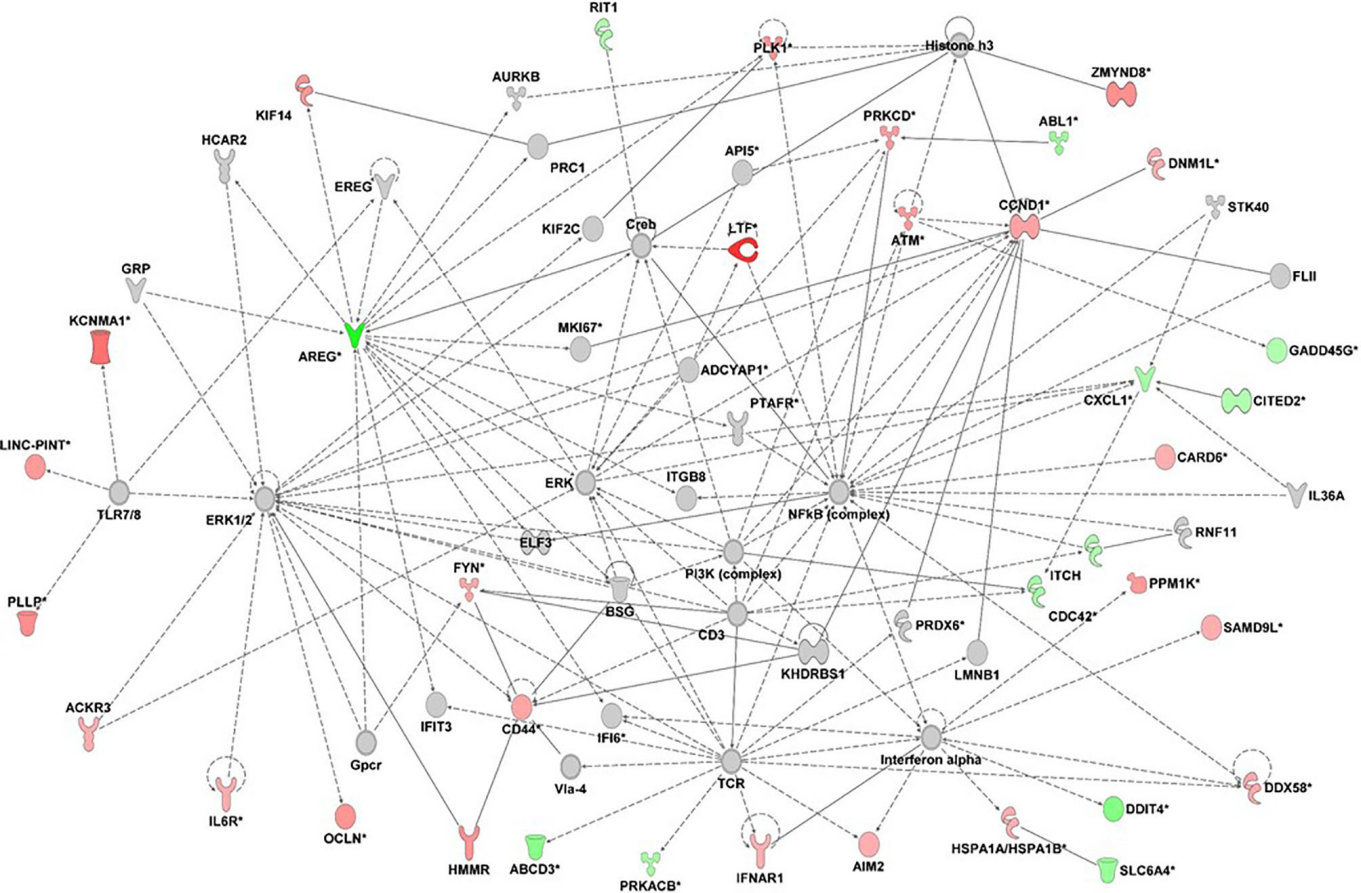
Gene network analysis derived from differentially expressed genes (fold change +/- 1.5) of 20 patients with type 1 diabetes in relation to 20 health controls (p<0.05). Red= up-regulated genes; green= downregulated genes; ::= direct interaction; — = indirect interaction. the graph was done with IPA-Ingenuity Pathways Analysis, Qiagen.

Unsupervised network analysis pointed towards enrichment of Connective Tissue Development and Function, Cellular Growth and Proliferation. The most important nodes/hubs were the *NF-kB* and *ERK*, followed by TCR and IFN-alpha, related to inflammation and host defense against bacteria and viruses. Among differentially expressed genes, the most connected were the upregulated *CCND1* and *ATM*, as well as the downregulated *AREG* and *CXCL1* genes (**Figure 4**).

Otherwise, genes associated with resolution of inflammation/Th2 response, immunomodulation (*SMAD6, AREG, CITED2, ITCH,TLE1*) were reduced, as well as those related to apoptosis and autophagy (*RIT1, DDIT4).*

The IPA feature Top Upstream regulator analysis indicates a gene whose effect overlaps with the tested gene profile, and identified YAP1 (yes-associated protein 1), a transcriptional regulator that activates the transcription of genes involved in cell proliferation and suppression of apoptosis (FC=2.62, P=0.0003).

**Figure 5:**
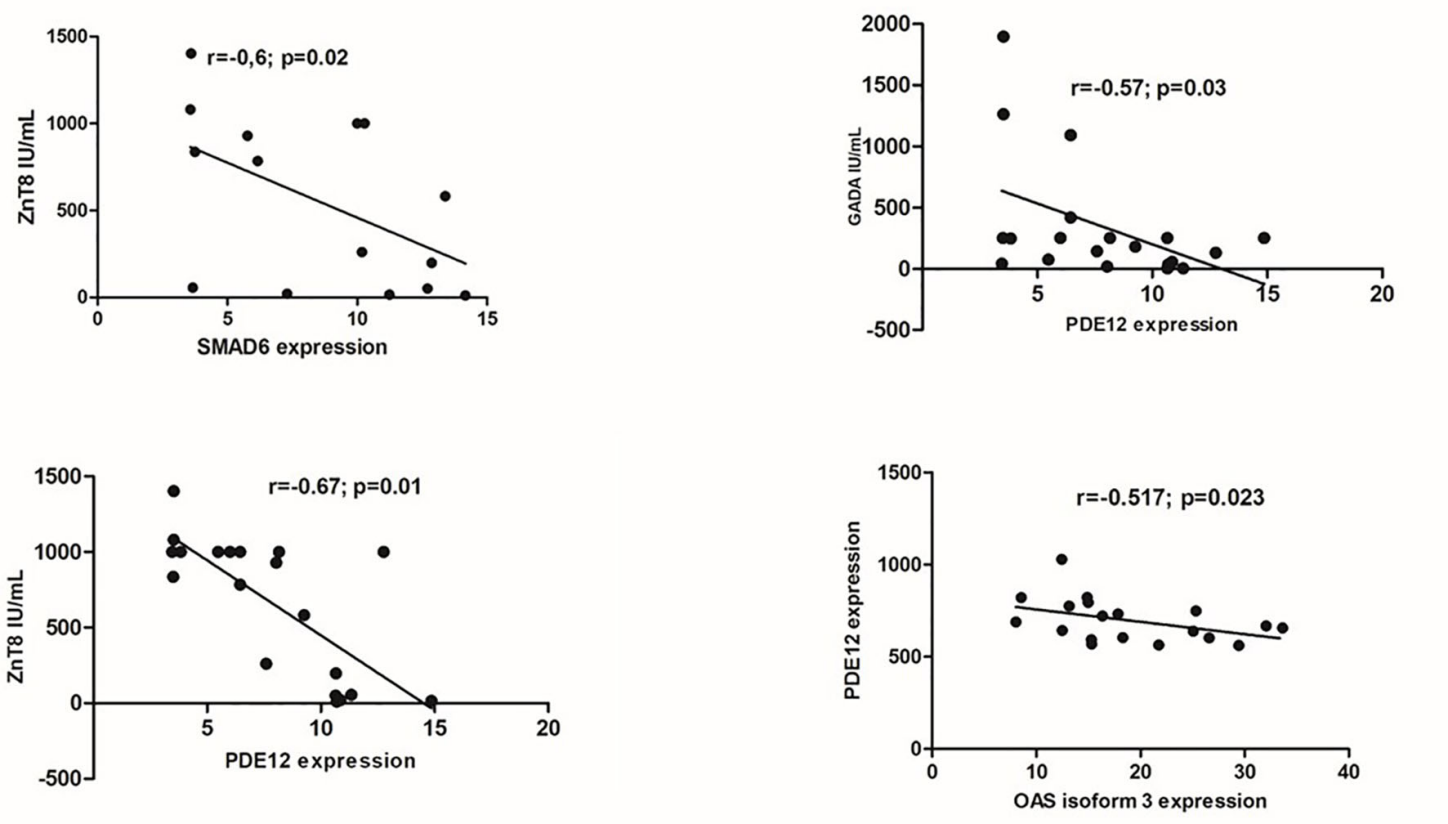
Correlations between gene expression (in peripheral blood mononuclear cells) and serum levels of anti-glutamic acid decarboxylase (GADA) and anti-zinc transporter 8 (ZnT8A) autoantibodies.; SMAD6- Mothers against decapentaplegic homolog 6; PDE12- Phosphodiesterase 12; OAS 3: 2’,5’-Oligoadenylate synthetase isoform 3.

The expressions of the major DEGs did not differ between groups considering sex, age and self-reported skin color. Principal component analysis based on the expression of the 346 DEGs across the 40 samples, according to their gender or age were also similar (**Supplementary Figure 1S**). In T1D group there were negative correlations of GAD65A and Znt8A levels with two down-regulated DEGs: GAD65A with PDE12(r=-0.57; p=0.03), ZnT8A with PDE12(r=-0.67; p=0.01) and SMAD6 (r=-0.60; p=0.02) and negative correlation of PDE12 with 2’,5’-Oligoadenylate synthetase isoform 3 enzyme (OAS3) (r=-0.517; p= 0.023), related to antiviral response (**Figure 5**).

Three of the upregulated genes (LTF, DEFA4 and CTSG) and two of downregulated genes (AREG and SMAD6) had their expression confirmed by quantitative real-time polymerase chain reaction qRT-PCR (**Table 3**), making our results more consistent.

**Table 3:** Gene expression validated by qRT-PCR.

Gene/Endogene	Relative gene expression in T1D patients	Relative gene expression in controls	Fold Change	P value	Mean(case)	Std. Deviation(case)	Std. Error of Mean (case)	Mean (control)	Std. Deviation (control)	Std. Error of Mean (control)
SMAD6/ACTB	0.38	0.63	-1.7	0.008	0,4084	0,1859	0,04157	1,908	2,175	0,4863
AREG/ ACTB	0.11	1.10	-10	0.002	0,9306	2,519	0,5632	3,401	4,222	0,9442
DEFA4/ ACTB	8.05	0.87	9.3	0.0002	15,09	22,56	5,175	2,261	3,041	0,6801
LTF/ ACTB	7.48	0.85	8.8	0.0002	17,35	30,62	7,024	3,099	5,911	1,322
CTSG/ ACTB	4.35	0.97	4.5	0.004	7,728	9,009	2,067	1,751	2,384	0,5331

Relative gene expression = 2^-ΔΔCt; median.

SMAD6, Mothers Against Decapentaplegic Homolog 6; AREG, Amphiregulin; CTSG, Cathepsin G; DEFA4, Defensin alpha 4; KCNMA1, Potassium Large Conductance Calcium-Activated Channel; LTF, Lactoferrin; ACTB, Actin Beta.

**Figure 6:**
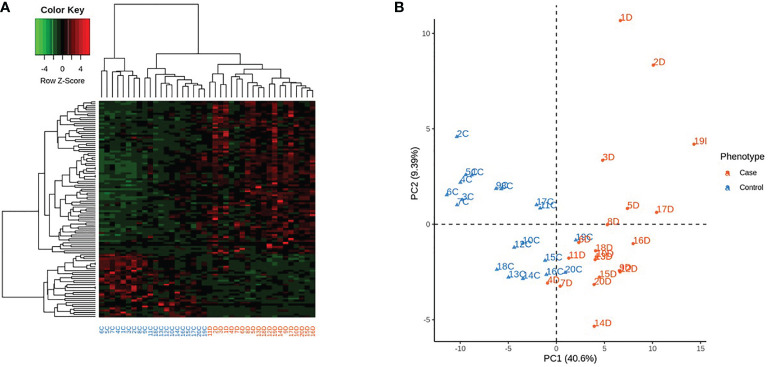
Expression of the 105 differentially expressed Long non coding RNAs across the 40 samples, according to their phenotype. Control identifiers are written in blue, and case identifiers in orange. **(A)** Complete-linkage hierarchical clustering (HCA) based on spearman correlation. **(B)** First two components of principal component analysis (PCA).

There were 105 lnc RNAs with differential expression between patients with T1D and controls (109 probes) (**Supplementary Table S5**). Seventy-four (70.5%) of lncRNAs were up regulated and 31 (29.5%) of them were downregulated. Heatmap and principal component analysis based on the expression of the 105 LncRNA across the 40 samples, confirmed the interest for these lncRNAs (**Figure 6**). These clusterings were not affected by gender or age (**Supplementary Figure 2**).

The LncTarD database evidenced the association of 31 of the LncRNAs with several oncologic and proliferative diseases (**Supplementary Table 6S**) (17). Some of these associations were predicted (n=28, - **Supplementary Table 7S**) and some of them have been experimentally confirmed (n=17, **Supplementary Table 8S**). Some of them were related to chronic diabetes complications like nephropathy and cardiomyopathy and to glucose levels (NEAT1, MALAT1, PVT1, HOTAIRM1) or to autoimmune diseases like rheumatoid arthritis and systemic lupus erythematosus, multiple sclerosis, polymyositis, psoriasis, autoimmune thyroid disease and T1D (MALAT1, NEAT1, PVT1). Others were associated to Type 2 diabetes (CASC15, LINC01127, NUTM2B-AS1) or diabetes renal injury in T1D (HOTAIRM1) (18).

In order to identify the targets of the differentially lnc RNAs we screened two databases (lncTarD and lncRNA2target). In the first one targets were described for the 7 following lnc RNAs (CASC15, FBXL19-AS1, HOTAIRM1, MALAT1, NEAT1,PVT1, RAD51-AS1) (19) (**Supplementary Table 9S**), whereas in the second one targets were identified for the 8 following lnc RNAs (CASC15, FBXL19-AS1, HOTAIRM1, LINC01410, MALAT1, NEAT1, PVT1, RAD51-AS1) (20) (**Supplementary Table 10S**). Target genes of MALAT and NEAT (HIF1A, NFKB1, miR-342-3p, miR-199b, SOCs1, CXCL5 were related to inflammatory response (**Supplementary Table 9S**). Information on the expression of these specific lncRNAs’ targets are provided in **Supplementary Table 9S** and in **Supplementary Table 10S**. Four targets are differentially expressed (HMMR, LAYN, OCLN and CCND1). These four differentially Targets are regulated by the same lncRNA MALAT1 which is overexpressed in patients. Among them, only LAYN is downregulated.

## Discussion

In this paper, we have shown that patients with recent-onset T1D displayed a higher number of upregulated protein-coding genes in peripheral PBMCs in comparison with healthy controls. Principal component analysis on mRNA and lncRNA clearly distinguished cases and controls. The IPA approach highlighted the involvement of 24 enriched pathways with two characteristic profiles: a developmental/metabolic profile and an inflammatory profile mainly associated with immune surveillance (**Figure 2**). The main pathways encompassed cell replication (nucleotide synthesis, DNA repair, cell cycle, endocytosis, cancer and metabolism) and cytokine signaling. Up-regulated genes participated in cell proliferation, migration and adhesion, defense against bacteria and virus, innate and adaptive immune response. Although these results were in accordance with the usual PBMCs role, they were much more prevalent in T1D and agree with the expected dysregulation of mononuclear cells in an immune-mediated disorder, especially in recent-onset T1D, a condition in which the autoimmune process is still highly active. Some were reported here for the first time. On the other hand, our data suggested that immunomodulation was depressed.

The highly proliferative process of the short-lived PBMCs and the increased immune activity require great energetic substrate supply. It had been probably provided from pathways enriched to replenish nutrients, metabolites and hormones like folate derivatives, methionine synthesis, amino acids catabolism and endocytic processes, besides DNA repair and genomic integrity pathways. In parallel, the DEGs present in interleukin IL-7 and Ephrin A (*erythropoietin-producing hepatocellular carcinoma*) signaling and in the activation of inflammasome (interferon inducible protein activated by viral DNA) pathways (**Supplementary Table 4S**), have been previously implicated in various facets of cell development, lymphocyte activation, immune surveillance and autoimmunity (21–23). The inflammasome activation and the IL-7 and ErbB2-ErbB3 pathways conferred susceptibility to other autoimmune diseases like SLE and psoriasis (21) or T1D (24) including in another Brazilian cohort (25). The activation of the HGF signaling *via* MET receptor, the Ephrin receptor and the STAT3 signaling pathways were recently related to the T1D pathogenesis mediated by increased serum levels of *miR-101-3p* by our group (26). In accordance with proinflammatory stimulation of immunity, the top four most differentially expressed genes in PBMC of T1D patients (**Figure 3**) were anti-microbial peptides, with pro-inflammatory activity (*DEFA1, DEFA4*, *LTF, CTSG*) (22). Likewise, KCNMA1 (Potassium Large Conductance Calcium-Activated Channel), another upregulated gene, was shown to be related to the NF-κB-dependent inflammatory response and T1D pathogenesis (27). Umnyakova et al. (22) suggested that autoimmune reactions could result from pro-inflammatory responses triggered by host anti-microbial peptides, particularly through the production of type I Interferon (IFN), an important trigger of autoimmune diseases. Non-specific immune stimulation causing persistent and low-grade inflammation can culminate in the loss of self-tolerance in the cases of pathogen-induced triggering of autoimmunity. In agreement, the level of neutrophil α-defensin (HNP-1-3) was increased in blood of patients with T1D and SLE and in synovial fluid of RA (22) and Cathepsin G, in several autoimmune diseases like dermatomyositis, scleroderma, systemic sclerosis, RA, SLE, Sjogren’s syndrome and T1D (28).

Zou et al. (29) showed that CATG degraded proinsulin in B cell (*in vitro*) and activated proinsulin reactive T cells. CATG activity was elevated in PBMC from T1D patients and revocation of its activity resulted in functional inhibition of proinsulin-reactive T cells. CATG small interfering RNA used in the pre-diabetic mellitus stage of non-obese diabetic mice improved the function of islet beta cells, reduced islet inflammation and beta cell apoptosis, and lowered the activation level of CD4+T cells, slowing down the progression of diabetes. Several other upregulated DEGs in our T1D group have been previously implicated in other inflammatory and autoimmune diseases like RA, SLE, MS, thyroiditis and T1D (27, 30–34).

Network analysis evidenced similar processes. The most important nodes/hubs according to the number of edges/connections were the *ERK*, *NF-kB* (nuclear factor kappa-light-chain-enhancer of activated B cells), T cell receptor (*TCR)* and *IFN-alpha* nodes, associated with several upregulated genes acting on cellular development, lymphocyte activation and immune responses (23) (**Figure 4**). A connection can be seem between *LTF* and *KCNMA1* inflammatory genes with NFKB and TLR7/8 nodes. TCR and interferon alpha nodes controls T cell effector response to foreign antigens, anti-viral defense and interferons production. Taking into account all over-expressed genes in our cohort, several of them are related to virus entrance, sensor of virus and anti-viral response as *AIM2, DDX58, TLR7, IFNAR1, DEFA4, CTSG, STIM2, CDC42, NEAT1,NAIP, KIF4A* (22, 23, 35, 36). A causal association between genes involved in host–virus interactions and susceptibility to T1D as well as to multiple other autoimmune disorders through an IFN signature pattern, was suggested (36). Further, it is important to note that transient upregulation of type I IFNs can be seen in genetically predisposed children preceding the seroconversion of T1D-related autoantibodies (7, 8).

So, the transcriptional signatures of PBMCs from our recent-onset T1D patients reflected inflammatory mechanisms common to various autoimmune diseases. The immune-mediated processes probably occurred in a glucose independent way, considering the absence of positive correlation of main gene expressions with fasting glucose levels and their involvement with several non-hyperglycemic autoimmune diseases.

On the other hand, there was a remarkable reduction in the expression of genes associated with immunomodulation and peripheral tolerance to self-antigens- like *AREG* (the most down-regulated gene in T1D) and *SMAD6*.

AREG (Amphiregulin) is a type II cytokine involved in tissue repair, cell survival, proliferation and motility, fomenting immune-mediated resistance and tolerance mechanisms. Through EFGR binding AREG promotes the immunosuppressive activity of Foxp3 regulatory T cells (Tregs) responsible for maintaining immune homeostasis and peripheral tolerance to self-antigens (37). Considering that AREG can restrain the antiviral activity of CD8+T cells in hepatitis B (38) we can suppose that its reduction could eventually be protective against virus infection, the potential candidate in T1D pathogenesis (36, 39, 40). The decreased expression of PDE12 in our T1D patients is in accordance with this. Inhibition of PDE12 may up-regulate the 2’,5’-Oligoadenylate synthetase (OAS) enzymes and RNase-L(OAS/RNase-L) pathway in response to viral infection resulting in increased resistance to a variety of viral pathogens (35). In accordance, there was a negative correlation of PDE12 and OAS isoform 3 (r=-0.517; p= 0.023) (**Figure 5**)

*SMAD6* suppresses the activation of p38 MAPK and JNK by facilitating the inhibition of TRAF6 (TNF receptor-associated factor 6) by TNF-α-induced protein A20, functioning as negative regulator of the NF-κB pathway. SMAD6 also suppresses innate immunity responses through inhibition of Toll-like receptors TLR4 and TLR2 signaling pathways (41). The lowered expression of the anti-inflammatory SMAD6 can also disturb the immunological tolerance in recent-onset T1D (42). The negative correlation of S*MAD*6 and *PDE12* expressions with ZNT8A and GADA levels agree with immunomodulation

In addition, we observed 105 LncRNAs with differential expression between patients with T1D and controls. They are defined as ncRNAs longer than 200 nucleotides unable to code proteins, located within the intergenic regions or overlapping antisense transcripts of protein coding genes. LncRNAs participate in various biological events and their dysregulation is involved in a variety of cellular processes and functions in cancer cell proliferation, apoptosis, metastasis, diabetes complications, but also in the pathogenesis of inflammatory and autoimmune diseases (43). Whereas the downregulation of lncRNA PVT1 seems to protect pancreatic β cells from injury (44), both up-regulated MALAT1 and NEAT1 had been related to inflammatory mediators (TNF-alpha, IL-6, CXCL10) (45), SLE pathogenesis (46) and innate immune response against viral infections (47). MALAT1 increased with hyperglycemia and was also implicated in islet beta cell dysfunction (48).

MALAT1 is among well studied and highly conserved lncRNAs, linked to a variety of pathological processes including various malignancies and diabetes-related complications. Through regulation of expression of genes related to motility and tissue barrier permeability like HMMR, LAYN, OCLN, CCND1, MALAT-1 seems to control metastasis and promote cell motility and growth (49–52).

MALAT1 can also play significant roles in pathophysiological processes, tissue inflammation, tumor progression, angiogenesis, cardiovascular remodeling, liver fibrosis, and diabetes progression by modulating gene transcription (53). MALAT1 is obviously an essential regulatory factor for ischemic reperfusion injury due to diabetes mellitus cerebrovascular diseases. In diabetic cataract, MALAT1 promote the apoptosis and oxidative stress of HLECs through the initiation of the p38MAPK signaling pathway (54), MALAT1 is considerably overexpressed in cardiac tissue of rats with diabetes, and its inhibition results in improvement in the cardiac function (55). Finally, MALAT1 was notably highly expressed in kidney tissues from C57BL/6 mice with streptozocin induced diabetic kidney disease (56). These results concluded that MALAT1 is a potential diagnostic and future targeted therapy for diabetes-associated complications.

The involvement of both LncRNAs (NEAT1, MALT1) in T1D pathogenesis is in accordance with the inflammatory and anti-viral response in our gene expression profile. Interesting, the MALAT-1 down-regulated LAYN gene is expressed in Treg cells under activation conditions (57), probably contributing to the immune aggression.

Considering all these data, we can conclude that the overall immune footprint in the peripheral blood expression pattern was a pro-inflammatory one with reduced expression of regulatory T cell circuit.

Previous transcriptome reports in T1D pointed to some divergences. Although the activation of immune system was strongly evidenced in antibody positive individual at risk for T1D (6, 14, 58), including an IFN-regulated signature consistent with the hypothesis of viral etiology (4, 7, 8), inflammatory pathways were observed in some (3, 5, 13) but not all (4, 14) studies in recent onset T1D. The inconsistencies between authors could come from multiple perturbations in pathways controlling cellular metabolism and survival, different immune stimulus acting on different genes, contributing to either a tolerogenic or effector inflammatory response, changing the autoimmune aggression with time (5).

Some of the upregulated inflammatory pathways potentially implicated in T1D pathogenesis were also reported to adopt, in some cases, an immunomodulatory effect, reducing the overstimulation of the immune system and sparing tissue damage (22), like HER2/HER3, HGF, CATG and LTF pathways. The same probably account for the increased AREG expression in other autoimmune diseases like SLE, Sjogren’s syndrome, and RA (59), contrary to our data. Despite not always elicited by the same genes, the common pathways in T1D and other autoimmune diseases point to proliferation, systemic inflammation and abnormalities of immune regulation over tolerance. Different environmental and genetic factors and the coverage of a great number of genes may have contributed to our unique observation of downregulation of *AREG* and *SMAD6*. On the other hand, all studies in individuals either at risk or with recent T1D were unanimous in confirming the increase in cell metabolism, biosynthesis of proteins, proliferation and immune cell cycle progression in T1D. However, it is necessary to observe that some enriched pathways in T1D patients may at least partially result from metabolic derangements that can modify lymphocytes metabolism, gene function and profile, causing mitochondrial dysfunction and DNA damage. Is still not clear how much of the increased expression of genes related to cell development and inflammation reflects active autoimmune reaction in pancreas, defense against pathogens or a process involving DNA damage and repair, elicited by metabolic disruption, gluco- and lipid toxicity present in T1D. Another point to consider is whether islet-infiltrating immune effectors really are in equilibrium with circulating cells. As interactions between pancreatic and circulating immune cells were supported by data of Cerosaletti K et al. (2) and Safari-Alighiarloo N et al. (3), we can argue that many changes observed in our cohort might have being accompanied by parallel changes in pancreatic –cells.

Our study had limitations. Our case series is not large, although it is similar in number to that of several other researchers (4, 8, 13, 33, 58). Our study has an additional strength because it evaluated the influence of clinical parameters such as age, sex on two groups with similar demographic data. We had data on skin color of each individual, however this parameter it is not specific enough to draw significant conclusions about the impact of ethnicity. Moreover, even this study lead to interesting data, it will be essential in the future to conduct a study on a larger population using single cell RNA-seq on PBMCs in order to confirm the genes, the isoforms that are differentially expressed between cases and controls and the cell populations associated. Besides corroborating previous work on Caucasians, it brings new data regarding the genetic diversity and admixture prevalent in Brazil.

## Conclusion

Our results expose the complexity of the autoimmune aggression in T1D comprising chronic activation of anti-infectious and inflammatory pathways, upregulation of innate immune response and commitment of immunomodulation. It also evidenced the increased metabolic demand associated with immune cell proliferation, differentiation and nutrition, bringing protective and inflammatory pathways that can be addressed to control the disease.

## Data Availability Statement

The data sets have been deposited in GEO public database under accession number GSE156035. They are also available in **Supplementary Material** or by the corresponding author upon request.

## Ethics Statement

The study was approved by the Research Ethical Committee of Hospital das Clinicas, Faculdade de Medicina, Universidade de Sao Paulo (Cappesq 11601), following guidelines in the Declaration of Helsinki. The patients/participants provided their written informed consent to participate in this study.

## Author Contributions

AS, CC, EC-N and MS designed the research. AS, NG, FB, and CM-F conducted the research. PB, AB, and CC performed statistical analyses. All authors contributed to the analysis of the data and reviewed the manuscript. AS, CC, EC-N, and MS finalized the manuscript.

## Funding

This study was supported by European Association for the Study of Diabetes (EASD) and São Paulo Research Foundation (FAPESP- Process number 2019/06664-4), non-profit foundations. This work was also supported by the Institut National de la Santé et de la Recherche Médicale (INSERM); the French Agency for Research (Agence Nationale de la Recherche-ANR (grant numbers: “Br-Fr-Chagas”, “landscardio”).

## Conflict of Interest

The authors declare that the research was conducted in the absence of any commercial or financial relationships that could be construed as a potential conflict of interest.

## Publisher’s Note

All claims expressed in this article are solely those of the authors and do not necessarily represent those of their affiliated organizations, or those of the publisher, the editors and the reviewers. Any product that may be evaluated in this article, or claim that may be made by its manufacturer, is not guaranteed or endorsed by the publisher.

## Abbreviations

LFT- Lactoferrin; DEFA4, Defensin, alpha 4; DEFA1, Defensin, alpha 1; CTSG, Cathepsin G; KCNMA1- Potassium Calcium-Activated Channel Subfamily M Alpha 1; AREG- Amphiregulin; SMAD6, Mothers Against Decapentaplegic Homolog 6; PDE12, Phosphodiesterase 12; MALAT1, Metastasis Associated Lung Adenocarcinoma Transcript 1; NEAT1, Nuclear Enriched Abundant Transcript 1; PVT1- Plasmacytoma Variant Translocation 1.
