# Supplementary figures and images for: Prevalence of Inflammatory Pathways Over Immuno-Tolerance in Peripheral Blood Mononuclear Cells of Recent-Onset Type 1 Diabetes
Source: Front Immunol. 2022 Jan 4;12:765264. doi: 10.3389/fimmu.2021.765264 (PMC8764313; doi:10.3389/fimmu.2021.765264)

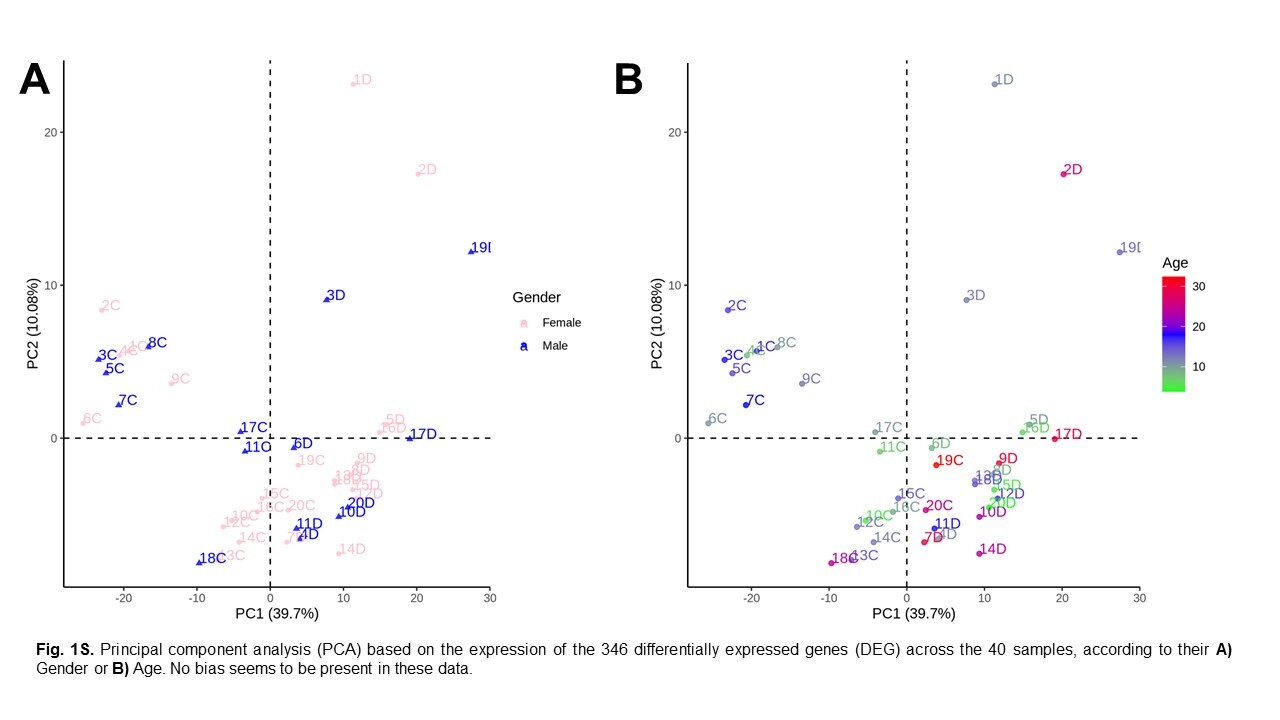

Supplement: Supplementary file 11 [file Image_1.jpg]

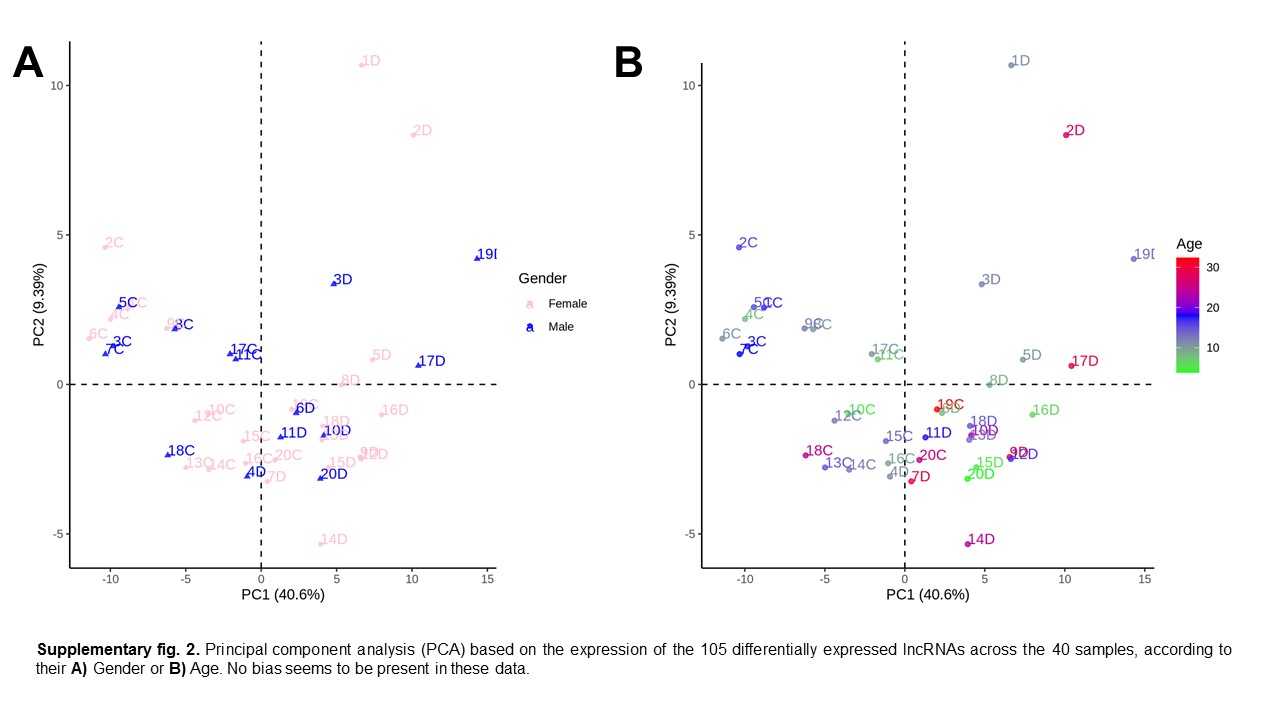

Supplement: Supplementary file 12 [file Image_2.jpg]
